# Cardiovascular Risk Factors of Adults Age 20–49 Years in the United States, 1971–2012: A Series of Cross-Sectional Studies

**DOI:** 10.1371/journal.pone.0161770

**Published:** 2016-08-23

**Authors:** Sarah S. Casagrande, Andy Menke, Catherine C. Cowie

**Affiliations:** 1 Public Health Research, Social & Scientific Systems, Inc., Silver Spring, Maryland, United States of America; 2 Division of Diabetes, Endocrinology, and Metabolic Diseases, National Institute of Diabetes and Digestive and Kidney Diseases, Bethesda, Maryland, United States of America; University of Bologna, ITALY

## Abstract

**Background:**

The health of younger adults in the U.S. has important public health and economic-related implications. However, previous literature is insufficient to fully understand how the health of this group has changed over time. This study examined generational differences in cardiovascular risk factors of younger adults over the past 40 years.

**Methods:**

Data were from 6 nationally representative cross-sectional National Health and Nutrition Examination Surveys (1971–2012; N = 44,670). Participants were adults age 20–49 years who self-reported sociodemographic characteristics and health conditions, and had examination/laboratory measures for hypertension, hyperlipidemia, diabetes, obesity, and chronic kidney disease. Prevalences of sociodemographic characteristics and health status were determined by study period. Logistic regression was used to determine the odds [odds ratio (OR), 95% confidence interval] of health conditions by study period: models adjusted only for age, sex, and race, and fully adjusted models additionally adjusted for socioeconomic characteristics, smoking, BMI, diabetes, and/or hypertension (depending on the outcome) were assessed.

**Results:**

Participants in 2009–2012 were significantly more likely to be obese and have diabetes compared to those in 1971–1975 (OR = 4.98, 3.57–6.97; OR = 3.49, 1.59–7.65, respectively, fully adjusted). Participants in 2009–2012 vs. 1988–1994 were significantly more likely to have had hypertension but uncontrolled hypertension was significantly less likely (OR = 0.67, 0.52–0.86, fully adjusted). There was no difference over time for high cholesterol, but uncontrolled high cholesterol was significantly less likely in 2009–2012 vs. 1988–1994 (OR = 0.80, 0.68–0.94, fully adjusted). The use of hypertensive and cholesterol medications increased while chronic kidney and cardiovascular diseases were relatively stable.

**Conclusions:**

Cardiovascular risk factors of younger U.S. adults have worsened over the past 40 years, but treatment for hypertension and high cholesterol has improved. The sub-optimal and worsening health in younger adults may have a substantial impact on health care utilization and costs, and should be considered when developing health care practices.

## Background

The health of younger adults in the U.S. has important implications for individual quality of life, public health, and the economy of the country[1]. There has been much focus on the health of older adults in the U.S. as baby boomers (born between 1946 and 1964) reach the age of 65 years [2]. However, understanding the health status of younger adults is important for preparing for the economic and health-related impact on the country as these individuals age. In particular, the rising prevalence of obesity is cause for concern. Roughly 60% of adults age 20–39 years and 75% of adults age 40–59 years are overweight or obese which poses significant health and economic risks [3]. Obesity negatively affects many bodily systems, including hypertension, high cholesterol, diabetes, and cardiovascular disease [4]. Not surprisingly, these morbidities come at a cost; in the U.S., the incremental per capita cost for obesity, as compared to normal weight individuals, was estimated to be 43% higher [5]. In addition, a previous study indicates that younger adults in the U.S. have more morbidity than other high-income peer countries. For example, compared to their counterparts in England, women age 35–49 years in the U.S. had a significantly higher prevalence of obesity (36.8% vs. 21.5%), diabetes (3.3% vs. 1.8%), heart attack (1.5% vs. 0.6%), hypertension (19.5% vs. 14.2%), and low high-density lipoprotein cholesterol (10.8% vs. 4.9%) compared to their counterparts in England; similar differences were shown for women age 18–34 years and for men [6].

It is relatively unknown how the health of younger adults in the U.S. has changed over time. A previous national U.S. study of middle aged adults (age 46–64 years) found that the overall health status of baby boomers was poorer compared to adults of the same age from a previous generation [7]. Compared to middle-aged adults from the previous generation, baby boomers were more likely to have obesity (38.7% vs. 29.4%), diabetes (15.5% vs. 12.0%), hypertension (43.0% vs. 36.4%) and high cholesterol (73.5% vs. 33.8%) and were less likely to be current smokers (21.3% vs. 27.6%). Past research on diabetes among U.S. birth cohorts demonstrated that the prevalence of diabetes rose by a factor of 4.9 between the birth cohorts of 1910–1919 and 1980–1989, thus, the prevalence of diabetes in the U.S. is rapidly increasing from one birth cohort to the next [8]. A study on the birth cohort effects of obesity among U.S. adults found that more recent birth cohorts had a higher prevalence of obesity for all age groups and, therefore, younger adults were experiencing a greater duration of obesity over their lifetime [9]. Finally, an Australian study found that Generation Xers (born 1966–1980) had a higher prevalence of obesity and diabetes compared to Baby Boomers (born 1946–1964) when they were age 25–44 years [10].

Despite this previous literature, no study has completed a comprehensive assessment of cardiovascular risk factors in younger U.S. adults and compared the health of these adults to their counterparts of previous generations. To fill this gap, we determined generational differences in the cardiovascular risk factors of U.S. adults age 20–49 years during six time periods over a 41 year time span using data from the National Health and Nutrition Examination Surveys, 1971–2012. Results from this study could be used as a resource for future research on the impact of future health economics and policies and will help health professionals appropriately prepare for future health care needs.

## Methods

The National Health and Nutrition Examination Survey (NHANES) is a stratified multistage probability cluster survey conducted in the non-institutionalized U.S. population [11–14]. Participants are interviewed in their home for basic demographic and health information and then scheduled for a visit at the mobile examination center (MEC) to complete physical examinations and to obtain biosamples. The sample included 44,670 non-pregnant adults age 20–49 years [n = 7,571 in NHANES I (1971–1975), n = 7,439 NHANES II (1976–1980), n = 10,163 NHANES III (1988–1994), n = 7,836 NHANES 1999–2004, n = 5,620 NHANES 2005–2008, n = 6,041 NHANES 2009–2012]. Between 1971 and 2012 the response rates for the interview ranged from 73% to 89%; for the examination, response rates ranged from 68% to 80%. Written informed consent was obtained from all participants and was approved by the National Center for Health Statistics Institutional Review Board. The NHANES is publicly available at: http://www.cdc.gov/nchs/nhanes/nhanes_questionnaires.htm.

### Household Measures

Participants self-reported sociodemographic characteristics, including age, sex, education, race or race/ethnicity, marital status, employment, and smoking status. In NHANES I and II, race was reported as “white,” “negro,” or “other;” Asian participants were categorized as “other” and Mexicans were categorized as “white” unless definitely known to be American Indian or other non-white race. In NHANES III,NHANES 1999–2004, NHANES 2005–2008, and NHANES 2009–2012, race/ethnicity was reported as “non-Hispanic white,” “non-Hispanic black,” “Hispanic,” or “Other.” For continuity across all NHANES study periods, race was aggregated as “white,” “black,” or “other.” Smoking status was only asked among participants age ≥25 years in NHANES I and II; analyses of smoking were limited to those age 25–49 years. Participants self-reported a previous diagnosis of hypertension and current use of hypertensive medication; self-report of high cholesterol and use of cholesterol medication was collected in NHANES III, NHANES 1999–2004, NHANES 2005–2008, and NHANES 2009–2012.

### Examination and Laboratory Measures

Height and weight were measured in all surveys to determine body mass index (BMI, kg/m^2^; overweight, BMI 25.0–29.9kg/m^2^; obese, BMI≥30kg/m^2^). Weight was measured with the participant wearing only underwear, a paper gown, and foam slippers. Blood pressure levels were only reported for NHANES III and NHANES 1999–2004, 2005–2008, and 2009–2012 and the methods used for measuring blood pressure were similar over time; readings in NHANES I and II were not comparable to the other survey periods due to methodological differences, including the collection of only one blood pressure reading [11, 12]. Uncontrolled hypertension was defined as a blood pressure level ≥140/90 mmHg, regardless of hypertensive medication use. Three blood pressure readings were taken in the arm after the participant was seated and rested quietly for 5 minutes and averaged. A calibrated mercury-gravity manometer was used to measure blood pressure. Ever having hypertension was defined as a blood pressure level ≥140/90 mmHg or current self-reported use of hypertensive medication. Total cholesterol was measured as part of a standard blood draw and uncontrolled high cholesterol was defined as total cholesterol ≥200mg/dL, regardless of cholesterol medication use [15–17]. Ever having high cholesterol was defined as total cholesterol ≥200mg/dL or current self-reported use of cholesterol medication. Self-reported hypertension and high cholesterol were not included as part of the definition of ever having these conditions in order to understand the contribution of medication use when compared to the uncontrolled definitions. Participants were considered to have been previously diagnosed with diabetes if they answered “yes” when asked whether a physician had ever told them that they had diabetes; the definition of diabetes included both type 1 and type 2 diabetes since type could not be differentiated in all surveys. Fasting plasma glucose (FPG) was measured among participants who had fasted 8 to < 24 hours prior to the blood draw in NHANES II, III, and 1999–2004, 2005–2008, and 2009–2012. A second definition of diabetes included self-report of a physician diagnosis or FPG ≥126mg/dL. FPG was calibrated according to NHANES documentation to compare estimates over time. Chronic kidney disease (CKD) was determined using the Chronic Kidney Disease Epidemiology Collaboration (CKD-EPI) equation which estimates glomerular filtration rate (eGFR) from serum creatinine based on age, sex, and race[18]. An eGFR<60 mL/min per 1.73m^2^ was considered indicative of CKD. The methods for obtaining and handling blood samples were similar over time. Further details of the examination and laboratory measures and quality control procedures can be found at: http://www.cdc.gov/nchs/nhanes/nhanes_questionnaires.htm. Participants self-reported cardiovascular disease (CVD), which included heart attack or heart failure. A summary of the definitions of health outcomes and the years reported is provided in S1 Table.

### Statistical Analysis

Prevalence (percent, standard error) of sociodemographic characteristics and health status were determined by study period. Regression analysis was used to test for trends over time with the variable of interest as the outcome and study period as the main independent variable. Logistic regression analysis [odds ratio (OR), 95% confidence interval (CI)] was conducted to determine the association between study periods and health outcomes (obesity, overweight, uncontrolled hypertension, ever had hypertension, uncontrolled high cholesterol, ever had high cholesterol, diabetes, CKD, or CVD). Five models were assessed for each outcome: (1) unadjusted for covariates, (2) adjusted for age, sex, and race, (3) additionally adjusted for education, marital status, and unemployment, (4) additionally adjusted for smoking, and (5) additionally adjusted for diabetes, BMI, and/or hypertension depending on the outcome. In order to illustrate the full magnitude of how cardiovascular disease conditions have changed over time, forest plots were constructed for each outcome using results from the logistic regression that were adjusted only for the non-modifiable factors of age, sex, and race. Models were then adjusted for potential modifiable explanatory variables to demonstrate factors that led to the temporal trends in health outcomes, above and beyond the changes in the distribution of demographic factors in the population; there was no evidence of collinearity since the highest variance inflation factor was 1.16. Given that outcome data were not consistently collected in all survey periods, the reference study period depended on the outcome; NHANES I was the reference for obesity, overweight, self-reported diabetes, and CVD; NHANES II was the reference for diabetes defined by self-report or FPG; NHANES III was the reference for ever and uncontrolled hypertension, ever and uncontrolled high cholesterol and CKD. Since smoking status was only determined among adults age ≥25 years in NHANES I and II, the logistic regression analyses that included smoking were restricted to adults age ≥25 years.

Analyses could not be stratified by smaller age groups due to the low prevalence of many health conditions; thus, all analyses included adults age ≤49 years and are referred to as “younger adults.” All analyses accounted for the cluster design and used sample weights that corrected for non-response by adjusting weights according to the age, sex, and socioeconomic distribution of the target population, so that estimates are representative of the non-institutionalized U.S. population (SUDAAN User’s Manual, Release 9.2, 2008; Research Triangle Institute).

## Results

### Characteristics of the Study Population

There were fewer younger adults age 20–35 years in 2009–2012 (52.3%) compared to 1971–1975 (59.5%) (Table 1). The prevalence of having less than a high school education significantly decreased from 26.1% in 1971–1975 to 16.4% in 2009–2012. The prevalence of unemployment trended upwards, with larger upticks in NHANES 1988–1994 and NHANES 2009–2012. More were married in 1971–1975 compared to 2009–2012 and the prevalence of current smoking significantly decreased over time.

**Table 1:** Characteristics [% (SE)] of NHANES cohorts among adults age 20–49 years, 1971–2012.

	NHANES I (1971–1975) (n = 7571)	NHANES II (1976–1980) (n = 7444)	NHANES III (1988–1994) (n = 10,171)	NHANES 1999–2004 (n = 7839)	NHANES 2005–2008 (n = 5626)	NHANES 2009–2012 (n = 6045)	p-value for trend
**Age**							<0.001
20–35	59.5 (1.01)	63.4 (0.69)	57.0 (1.09)	50.7 (0.97)	50.7 (0.96)	52.3 (1.46)	
36–49	40.5 (1.01)	36.6 (0.69)	43.0 (1.09)	49.3 (0.97)	49.3 (0.96)	47.7 (1.46)	
**Sex**							0.055
Men	47.8 (0.60)	48.3 (0.58)	49.2 (0.55)	49.2 (0.64)	49.6 (0.61)	49.3 (0.65)	
Women	52.2 (0.60)	51.7 (0.58)	50.8 (0.55)	50.8 (0.64)	50.4 (0.61)	50.7 (0.65)	
**Race**							
White	88.2 (0.90)	86.6 (1.53)	79.5 (1.28)	82.6 (1.15)	81.2 (1.72)	78.9 (1.76)	<0.001
Black	10.7 (0.89)	10.8 (1.19)	12.1 (0.67)	12.2 (1.06)	12.6 (1.38)	12.4 (1.24)	0.141
Other	1.2 (0.19)	2.6 (0.87)	8.4 (0.88)	5.3 (0.56)	6.26 (0.75)	8.65 (0.89)	<0.001
**Education**							
< High school	26.1 (0.91)	21.8 (0.90)	18.5 (0.92)	17.9 (0.67)	17.9 (1.18)	16.4 (1.04)	<0.001
High school	38.6 (0.96)	35.6 (1.14)	34.4 (0.99)	25.8 (0.89)	23.8 (0.89)	20.3 (1.05)	<0.001
Some college	20.0 (0.80)	23.9 (0.76)	24.1 (0.77)	31.6 (0.89)	32.0 (0.73)	33.3 (1.12)	<0.001
College grad or above	15.3 (0.92)	18.7 (1.01)	22.9 (0.98)	24.7 (1.19)	26.3 (1.69)	30.0 (1.61)	<0.001
**Unemployed**	2.0 (0.21)	2.5 (0.21)	4.3 (0.31)	2.5 (0.23)	2.3 (0.23)	4.8 (0.33)	<0.001
**Marital Status**							
Married	75.6 (0.90)	75.3 (0.96)	59.6 (1.23)	54.1 (1.11)	53.1 (1.15)	49.0 (1.36)	<0.001
Widowed	1.5 (0.22)	3.1 (0.21)	0.55 (0.09)	0.63 (0.10)	0.44 (0.11)	0.66 (0.13)	<0.001
Divorced/separated	7.1 (0.42)	10.2 (0.32)	11.2 (0.56)	11.6 (0.60)	10.4 (0.53)	10.5 (0.66)	0.001
Living w/partner	^a^	^a^	5.5 (0.47)	7.9 (0.52)	11.1 (0.76)	11.1 (0.61)	<0.001
Never married	15.9 (0.88)	11.5 (0.88)	23.0 (1.03)	25.8 (1.09)	25.0 (1.25)	28.8 (1.77)	<0.001
**Smoking**^b^							
Current	44.8 (1.32)	41.9 (0.65)	33.4 (0.89)	30.0 (0.93)	28.0 (1.26)	23.8 (1.00)	<0.001
Former	20.0 (1.30)	18.7 (0.46)	18.6 (0.78)	16.7 (0.69)	16.8 (0.69)	15.8 (0.90)	0.001
Never	35.2 (1.36)	39.5 (0.59)	48.0 (0.92)	53.3 (1.15)	55.2 (1.23)	60.5 (0.32)	<0.001
**Obesity**^c^	12.2 (0.50)	12.6 (0.46)	19.9 (0.74)	29.4 (0.72)	32.1 (1.26)	33.4 (1.05)	<0.001
**Overweight**^d^	29.5 (0.70)	27.8 (0.76)	30.0 (0.71)	32.4 (0.88)	32.5 (0.85)	31.7 (0.90)	<0.001
**Hypertension**							
BP≥140/90mmHg	^e^	^e^	7.5 (0.39)	9.0 (0.54)	8.0 (0.44)	7.0 (0.42)	0.133
On medication	2.2 (0.22)	4.0 (0.26)	3.7 (0.36)	6.2 (0.36)	7.6 (0.55)	7.7 (0.52)	<0.001
Self-report	5.6 (0.36)	15.2 (0.58)	14.6 (0.67)	14.8 (0.57)	16.5 (0.96)	15.3 (0.71)	<0.001
**High cholesterol**							
≥200mg/dL	49.3 (0.90)	45.6 (0.96)	40.5 (1.26)	42.3 (0.66)	40.7 (0.89)	38.4 (0.74)	<0.001
On medication	^f^	^f^	1.1 (0.20)	3.5 (0.32)	5.1 (0.42)	4.8 (0.49)	<0.001
Self-report	^f^	^f^	13.2 (0.60)	15.9 (0.63)	18.4 (0.69)	17.1 (0.75)	<0.001
**Diabetes**							
Self-report only	0.89 (0.14)	1.4 (0.12)	1.9 (0.23)	2.8 (0.24)	3.4 (0.33)	3.2 (0.34)	<0.001
Self-report or FPG≥126 mg/dL		2.5 (0.28)	3.0 (0.29)	4.0 (0.43)	4.5 (0.39)	4.6 (0.44)	<0.001
**Chronic Kidney Disease**^g^	^h^	^h^	0.35 (0.10)	0.61 (0.11)	0.49 (0.14)	0.54 (0.14)	0.249
**Cardiovascular Disease**^h^	0.91 (0.13)	0.58 (0.09)	0.91 (0.15)	1.0 (0.13)	1.2 (0.13)	1.2 (0.19)	0.008

^a^ Data not collected in NHANES I or NHANES II

^b^Smoking among adults age 25–49

^c^ BMI≥30.0kg/m^2^

^d^ BMI 25.0–29.9kg/m^2^

^e^Reliable blood pressure measures were not available in NHANES I or NHANES II

^f^ Data not collected in NHANES I or NHANES II

^g^ Estimated glomerular filtration rate <60 mL/min per 1.73m^2^ based on the Chronic Kidney Disease Epidemiology Collaboration Equation, information not collected in NHANES I or NHANES II

^h^ Self-report.

The prevalence of obesity almost tripled between 1971–1975 and 2009–2012, while overweight increased less dramatically (Table 1). Hypertension (blood pressure ≥140/90mmHg) was stable between 1988 and 2012 and ranged from 7.0% to 9.0%; however, the prevalence of younger adults who reported taking hypertensive medications increased over three-fold between 1971–1975 and 2009–2012. High cholesterol (total cholesterol ≥200mg/dL) significantly decreased between 1971–1975 and 2009–2012, but the prevalence of cholesterol medication use increased over four-fold from 1.1% in 1988–1994 to 4.8% in 2009–2012. Diabetes significantly increased over the past 40 years with 0.9% self-reporting the disease in 1971–1975 and 3.2% in 2009–2012. CKD remained stable over time and the prevalence of CVD increased slightly from 0.9% in 1971–1975 to 1.2% in 2009–2012.

### Temporal Trends in Health Outcomes Adjusted for Age, Sex, and Race

**Fig 1:**
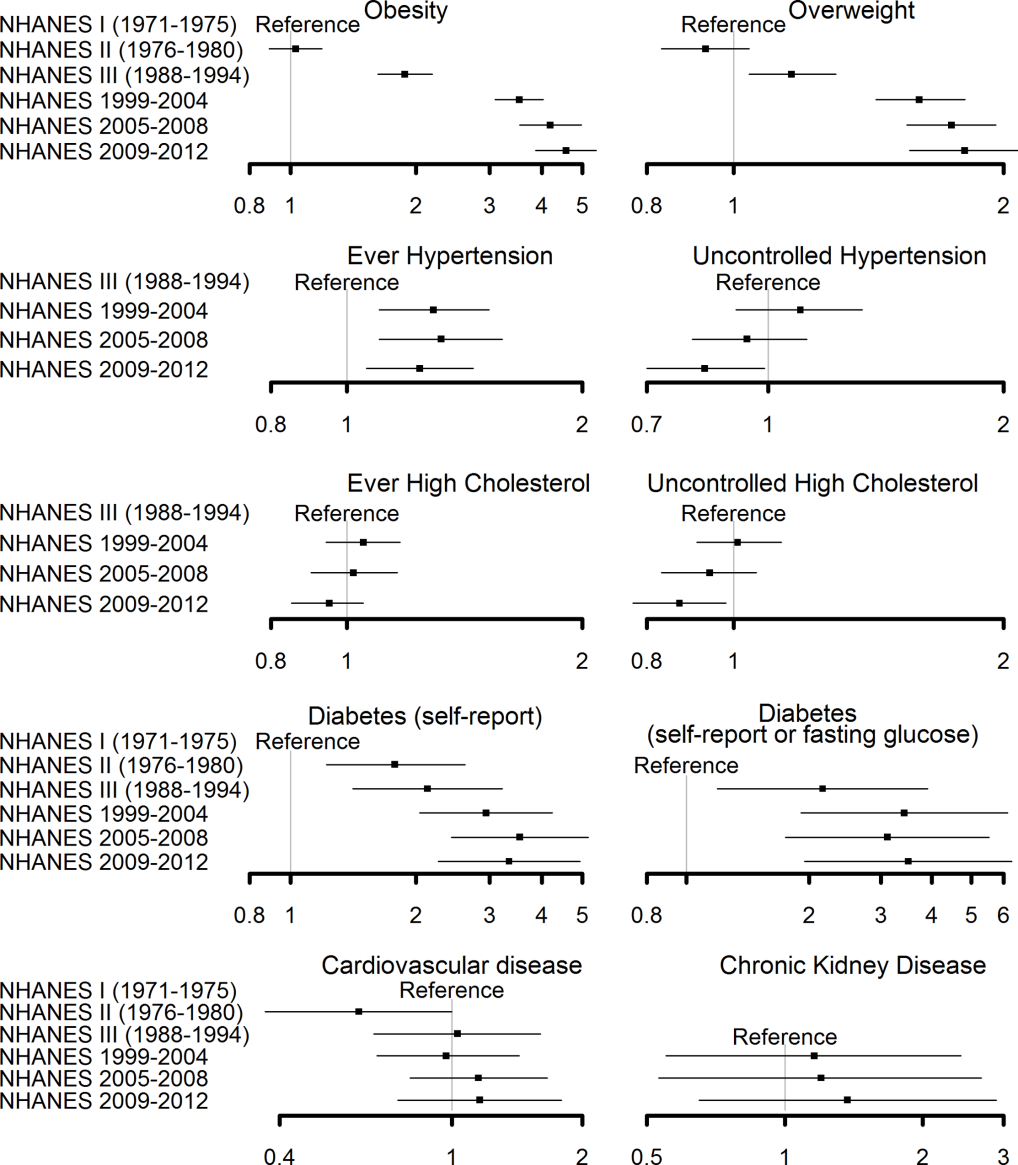
Forest plots (OR, 95% CI) for temporal trends in health outcomes among adults age 20–49 years, 1971–2012. Estimates are adjusted for age, sex, and race. Reference period is NHANES I for obesity, overweight, diabetes defined by self-report, and CVD; NHANES II for diabetes defined by self-report or FPG; NHANES III for ever hypertension, uncontrolled hypertension, ever high cholesterol, uncontrolled high cholesterol, and CKD.

To provide a public health perspective of how health outcomes have changed over time, Fig 1 shows the full magnitude of associations between study period and health outcomes when adjusting only for non-modifiable risk factors (age, sex, and race) among adults age 20–49 years (S2 Table). Younger adults were significantly more likely to be obese or overweight in 2009–2012 vs. 1971–1975 (OR = 4.57, 3.86–5.40; OR = 1.81, 1.57–2.09, respectively). Adults age 20–49 years were more likely to have ever had hypertension in 2009–2012 vs. 1988–1994 but were less likely to have uncontrolled hypertension. There was no association between study period and ever having high cholesterol but adults age 20–49 years were significantly less likely to have uncontrolled high cholesterol in 2009–2012 vs. 1988–1994. Diabetes, defined by self-report or additionally by FPG, was significantly higher in 2009–2012 vs. 1971–1975. There was no association between study period and CKD or CVD.

### Temporal Trends in Health Outcomes Adjusted for Explanatory Factors

#### Obesity

Adults age 25–49 years in 2009–2012 were significantly more likely to be obese (vs. normal weight) compared to those in 1971–1975 (OR = 4.98, 3.57–6.97, fully adjusted model) (Table 2). This relationship was also significant in the other study periods, except for 1976–1980, compared to 1971–1975.

**Table 2:** Odds ratio (95% confidence interval) of health outcomes associated with study period among adults age 25–49 years, 1971–2012.

	Model 1	Model 2	Model 3	Model 4	Model 5
	Unadjusted	Adjusted for age, race, sex	Model 2 + education, marital status, unemployment	Model 3 + smoking	Model 4 + diabetes^a^ or BMI^b^ or diabetes, BMI, and hypertension^c^
**Obesity**^d^					
NHANES I (1971–1975)	1.00	1.00	1.00	1.00	
NHANES II (1976–1980)	0.99 (0.85, 1.16)	1.02 (0.87, 1.19)	1.05 (0.90, 1.23)	1.04 (0.76, 1.44)	
NHANES III (1988–1994)	**1.75 (1.48, 2.06)**	**1.82 (1.53, 2.17)**	**2.03 (1.72, 2.41)**	**1.92 (1.38, 2.66)**	
NHANES 1999–2004	**3.35 (2.89, 3.87)**	**3.30 (2.82, 3.86)**	**3.90 (3.34, 4.55)**	**3.49 (2.53, 4.82)**	
NHANES 2005–2008	**3.91 (3.28, 4.67)**	**3.93 (3.26, 4.73)**	**4.62 (3.87, 5.52)**	**4.31 (3.09, 6.01)**	
NHANES 2009–2012	**4.32 (3.60, 5.18)**	**4.42 (3.67, 5.31)**	**5.43 (4.54, 6.48)**	**4.98 (3.57, 6.97)**	
**Overweight**^e^					
NHANES I (1971–1975)	1.00	1.00	1.00	1.00	
NHANES II (1976–1980)	0.93 (0.83, 1.05)	0.95 (0.84, 1.08)	0.97 (0.86, 1.09)	0.86 (0.71, 1.05)	
NHANES III (1988–1994)	1.12 (0.99, 1.26)	**1.14 (1.01, 1.29)**	**1.22 (1.08, 1.38)**	1.03 (0.82, 1.28)	
NHANES 1999–2004	**1.61 (1.42, 1.82)**	**1.59 (1.40, 1.81)**	**1.74 (1.53, 1.98)**	**1.44 (1.16, 1.80)**	
NHANES 2005–2008	**1.72 (1.52, 1.95)**	**1.74 (1.52, 1.98)**	**1.93 (1.69, 2.21)**	**1.59 (1.29, 1.96)**	
NHANES 2009–2012	**1.77 (1.54, 2.03)**	**1.86 (1.60, 2.15)**	**2.11 (1.82, 2.44)**	**1.69 (1.36, 2.11)**	
**Ever Hypertension**^**f**^					
NHANES III (1988–1994)	1.00	1.00	1.00	1.00	1.00^b^
NHANES 1999–2004	**1.44 (1.23, 1.68)**	**1.29 (1.10, 1.52)**	**1.33 (1.14, 1.55)**	**1.30 (1.03, 1.63)**	1.13 (0.90, 1.41)
NHANES 2005–2008	**1.48 (1.25, 1.76)**	**1.30 (1.09, 1.56)**	**1.34 (1.13, 1.59)**	**1.51 (1.17, 1.96)**	1.27 (0.99, 1.64)
NHANES 2009–2012	**1.37 (1.15, 1.63)**	**1.24 (1.05, 1.46)**	**1.28 (1.09, 1.50)**	**1.36 (1.08, 1.70)**	1.09 (0.86, 1.38)
**Uncontrolled Hypertension**^**g**^					
NHANES III (1988–1994)	1.00	1.00	1.00	1.00	1.00^b^
NHANES 1999–2004	**1.20 (1.02, 1.42)**	1.10 (0.92, 1.31)	1.13 (0.96, 1.33)	1.08 (0.86, 1.35)	0.97 (0.77, 1.22)
NHANES 2005–2008	1.02 (0.87, 1.21)	0.91 (0.77, 1.08)	0.93 (0.79, 1.09)	1.04 (0.81, 1.34)	0.92 (0.72, 1.19)
NHANES 2009–2012	0.90 (0.76, 1.08)	**0.82 (0.69, 0.98)**	**0.84 (0.70, 0.99)**	0.80 (0.63, 1.01)	**0.67 (0.52, 0.86)**
**Ever High Cholesterol**^**h**^					
NHANES III (1988–1994)	1.00	1.00	1.00	1.00	1.00^b^
NHANES 1999–2004	1.12 (1.00, 1.25)	1.05 (0.93, 1.17)	1.07 (0.95, 1.19)	1.03 (0.87, 1.21)	0.98 (0.83, 1.15)
NHANES 2005–2008	1.11 (0.98, 1.25)	1.03 (0.91, 1.16)	1.05 (0.93, 1.18)	1.07 (0.91, 1.26)	1.00 (0.85, 1.17)
NHANES 2009–2012	1.00 (0.90, 1.12)	0.94 (0.85, 1.05)	0.97 (0.87, 1.08)	0.98 (0.84, 1.15)	0.89 (0.76, 1.05)
**Uncontrolled High Cholesterol**^**i**^					
NHANES III (1988–1994)	1.00	1.00	1.00	1.00	1.00^b^
NHANES 1999–2004	1.07 (0.95, 1.20)	1.01 (0.90, 1.13)	1.02 (0.91, 1.15)	0.97 (0.82, 1.14)	0.93 (0.79, 1.10)
NHANES 2005–2008	1.00 (0.88, 1.13)	0.93 (0.82, 1.05)	0.95 (0.84, 1.07)	0.93 (0.80, 1.08)	0.88 (0.76, 1.02)
NHANES 2009–2012	0.91 (0.81, 1.02)	**0.86 (0.77, 0.96)**	**0.88 (0.78, 0.99)**	0.87 (0.74, 1.02)	**0.80 (0.68, 0.94)**
**Diabetes (self-report)**					
NHANES I (1971–1975)	1.00	1.00	1.00	1.00	1.00^b^
NHANES II (1976–1980)	**1.70 (1.13, 2.55)**	**1.81 (1.20, 2.74)**	**1.83 (1.21, 2.77)**	1.66 (0.77, 3.55)	1.68 (0.77, 3.66)
NHANES III (1988–1994)	**2.18 (1.41, 3.38)**	**2.23 (1.43, 3.49)**	**2.28 (1.45, 3.59)**	**2.79 (1.30, 6.02)**	**2.45 (1.12, 5.36)**
NHANES 1999–2004	**3.31 (2.24, 4.89)**	**3.08 (2.07, 4.58)**	**3.06 (2.05 (4.57)**	**3.76 (1.79, 7.87)**	**2.83 (1.33, 6.00)**
NHANES 2005–2008	**4.11 (2.75, 6.14)**	**3.74 (2.49, 5.63)**	**3.89 (2.59, 5.83)**	**4.61 (2.18, 9.75)**	**3.39 (1.59, 7.24)**
NHANES 2009–2012	**3.71 (2.47, 5.56)**	**3.43 (2.27, 5.19)**	**3.55 (2.32, 5.43)**	**4.85 (2.24, 10.49)**	**3.49 (1.59, 7.65)**
**Diabetes (self-report or FPG)**					
NHANES II (1976–1980)	1.00	1.00	1.00	1.00	1.00^b^
NHANES III (1988–1994)	**2.21 (1.22, 4.00)**	**2.15 (1.18, 3.90)**	**2.18 (1.21, 3.94)**	**2.54 (1.12, 5.76)**	2.11 (0.88, 5.09)
NHANES 1999–2004	**3.77 (2.10, 6.75)**	**3.29 (1.83, 5.93)**	**3.26 (1.82, 5.83)**	**4.14 (1.88, 9.12)**	**2.88 (1.27, 6.52)**
NHANES 2005–2008	**3.70 (2.11, 6.48)**	**3.10 (1.74, 5.53)**	**3.20 (1.80, 5.68)**	**3.65 (1.62, 8.24)**	**2.41 (1.05, 5.51)**
NHANES 2009–2012	**3.72 (2.08, 6.67)**	**3.28 (1.83, 5.87)**	**3.37 (1.88, 6.03)**	**4.39 (1.95, 9.87)**	**2.83 (1.22, 6.58)**
**Chronic Kidney Disease**^j^					
NHANES III (1988–1994)	1.00	1.00	1.00	1.00	1.00^a^^,^^b^
NHANES 1999–2004	1.22 (0.56, 2.68)	1.02 (0.46, 2.26)	0.93 (0.41, 2.09)	0.62 (0.24, 1.59)	0.61 (0.23, 1.59)
NHANES 2005–2008	1.49 (0.66, 3.37)	1.25 (0.56, 2.82)	1.14 (0.49, 2.65)	0.70 (0.23, 2.18)	0.67 (0.21, 2.13)
NHANES 2009–2012	1.65 (0.77, 3.53)	1.44 (0.68, 3.05)	1.31 (0.60, 2.84)	1.48 (0.53, 4.15)	1.30 (0.44, 3.87)
**Cardiovascular Disease**^k^					
NHANES I (1971–1975)	1.00	1.00	1.00	1.00	1.00^c^
NHANES II (1976–1980)	**0.56 (0.34, 0.93)**	0.60 (0.37, 0.99)	0.61 (0.37, 1.02)	1.20 (0.49, 2.95)	0.93 (0.38, 2.26)
NHANES III (1988–1994)	0.94 (0.60, 1.47)	1.01 (0.64, 1.57)	1.14 (0.72, 1.82)	2.34 (0.97, 5.67)	1.31 (0.54, 3.15)
NHANES 1999–2004	0.98 (0.66, 1.46)	0.91 (0.61, 1.35)	1.03 (0.67, 1.58)	2.37 (1.00, 6.61)	1.33 (0.56, 3.17)
NHANES 2005–2008	1.20 (0.83, 1.75)	1.09 (0.75, 1.58)	1.24 (0.85, 1.81)	**3.24 (1.42, 7.36)**	1.57 (0.68, 3.63)
NHANES 2009–2012	1.21 (0.79, 1.86)	1.14 (0.74, 1.75)	1.30 (0.82, 2.06)	**3.64 (1.50, 8.81)**	1.88 (0.75, 4.70)

Boldface indicates statistical significance (p<0.05)

^a^ Additionally adjusted for diabetes

^b^ Additionally adjusted for BMI

^c^ Additionally adjusted for diabetes, BMI, and hypertension

^d^ BMI≥30.0kg/m^2^ vs. BMI 18.5-<25.0 kg/m^2^

^e^ BMI 25.0–29.9kg/m^2^ vs. BMI 18.5- <25.0kg/m^2^

^f^ Ever hypertension defined as self-reported hypertensive medication or BP≥140/90 mmHg

^g^ Hypertension defined as BP≥140/90 mmHg

^h^ Ever high cholesterol defined as self-reported cholesterol medication or ≥200mg/dL

^i^High cholesterol defined as ≥200mg/dL

^j^ Estimated glomerular filtration rate <60 mL/min per 1.73m^2^ based on the Chronic Kidney Disease Epidemiology Collaboration Equation

^k^ CVD is self-reported.

#### Overweight

Adults age 25–49 years were significantly more likely to be overweight (vs. normal weight) in 2009–2012 compared to their counterparts in 1971–1975 (OR = 1.69, 1.36–2.11, fully adjusted model) (Table 2). This relationship was also significant for younger adults in 1999–2004 and 2005–2008 vs. 1971–1975.

#### Hypertension

Adults age 25–49 years were significantly more likely to have ever had hypertension in 2009–2012 vs. 1988–1994 in all models except for the fully adjusted model that included BMI as a covariate (OR = 1.36, 1.08–1.70, adjusted for sociodemographics and smoking) (Table 2). These associations were also present in 2005–2008 and 1999–2004 vs. 1988–1994. These results suggest that the increase in BMI had a large effect on the increase in ever having hypertension over time.

Adults age 25–49 years in 2009–2012 were significantly less likely to have uncontrolled hypertension compared to their counterparts in 1988–1994 (OR = 0.67, 0.52–0.86, fully adjusted model). There was a steady decrease in the OR as explanatory covariates were added to the model. Although the association did not always reach statistical significance in other models, there was limited power to detect an association of the magnitude observed in the intermediate results.

#### High Cholesterol

There were no significant associations between study period and ever having high cholesterol (Table 2). Adults age 25–49 years in 2009–2012 were significantly less likely to have uncontrolled high cholesterol compared to their counterparts in 1988–1994 (OR = 0.80, 0.68–0.94, fully adjusted model). The magnitude of the association was consistent across other models and, although the association did not always reach statistical significance, there was limited power to detect an association of the magnitude observed in the intermediate results.

#### Diabetes

Adults age 25–49 years in 2009–2012 were significantly more likely to have self-reported diabetes compared to those in 1971–1975 (OR = 3.49, 1.59–7.65, fully adjusted model); this relationship persisted in all study periods except 1976–1980 (Table 2). Results were similar in significance and magnitude when defining diabetes by self-report or FPG.

#### Chronic Kidney Disease

There were no significant associations between study period and CKD (OR = 1.30, 0.44–3.87, in 2009–2012 vs. 1988–1994, fully adjusted model) (Table 2).

#### Cardiovascular Disease

After adjusting for demographics, sociodemographics, and smoking status, adults age 25–49 years in 2005–2008 and 2009–2012 were significantly more likely to have CVD compared to those in 1971–1975 (Table 2). However, after adjustment for hypertension, diabetes, and BMI, the association became non-significant.

## Discussion

Cardiovascular risk factors in younger adults have worsened over the past 40 years. Younger adults in recent cohorts were more likely to have diabetes, obesity, and hypertension, and there was little change in ever having high cholesterol compared to their counterparts of previous generations, even after adjustment for other characteristics that have changed over time. Coincident with these findings, a greater percentage of younger adults in recent cohorts were taking hypertensive and cholesterol medications, which have resulted in better control of these conditions. CVD prevalence was relatively stable which may be due to reductions in smoking being offset by increases in obesity, hypertension, and diabetes. Younger adulthood is a formative time that has a significant impact on future health behaviors and employment potential. Morbidity during this age period can significantly affect life expectancy, quality of life and economic well-being [19].

To complement these results, there is evidence that successive birth cohorts are developing obesity earlier. Results from a national U.S. study between 1971 and 2006 indicated that obesity was occurring at earlier ages in more recent birth cohorts and, consequently, the duration of obesity for younger adults was greater over their lifetime [9]. The effect of young onset obesity and greater duration of disease has multiple implications for additional comorbidities such as hypertension, high cholesterol, diabetes, and cardiovascular disease [4, 20].

Previous studies have found similar trends in cardiovascular risk factors, although there are few studies that have assessed temporal trends over several decades and have limited the analysis to adults ≤49 years as was done in the current study. In the U.S., there is supportive evidence from NHANES that the use of hypertensive and cholesterol medications has increased and blood pressure and cholesterol control has improved among adults age≥18 years [21, 22]. Better control is likely due to the effectiveness of medications for these conditions. Similar to our findings, it is well documented that obesity and diabetes have significantly increased in the U.S. and worldwide, especially in high and middle income countries, to epidemic levels [23, 24]. However, these are multifactorial diseases and, therefore, difficult to epidemics to curb; genetics, public policies, food and built environments, health systems, and individual behaviors are all relevant factors [25, 26]. In the current study, we were able to account for many explanatory variables, as discussed below, but could not account for factors such as stress, social structures and support, public policies, and dietary and physical activity patterns [26–29].

Changes in certain sociodemographic factors may have influenced the poorer health outcomes that were observed over the study period. First, the lingering effects of recessions and unemployment since the late 1980’s may have translated to lost wages and, consequently, to less access to health care and less money to purchase nutritious food; these changes would influence the prevalence of obesity and diabetes[30]. Previous work has also shown that obesity is linked to long-term unemployment [31]. Second, fewer younger adults were married in 2009–2012 and, although studies have reported varied results, there is some evidence that marriage can have a positive impact on health [32–36]. However, the happiness of a marriage may be an important factor; one study found that spouses in satisfying relationships relaxed their efforts to maintain their weight while those in less satisfying relationships were more likely to consider divorce and less likely to gain weight [37]. Despite adjusting for these sociodemographic characteristics, we still found strong associations with obesity and diabetes over time; thus, there are other factors or aspects to these factors that are influencing these associations. The relationship between sociodemographic factors and health is complex, both at the individual and public level, and deserves further investigation among this population.

Changes in the physical environment may influence inactivity and poor eating and, consequently, be associated with the increase in prevalence of obesity and diabetes that was observed over these study periods [38, 39]. The modern environment has food that is nearly always available, is often processed, and physical activity is rarely required to obtain it which makes energy balance difficult to achieve. During the time period covered in this study, the U.S. has experienced significant urban sprawl which leads to fewer opportunities to walk for transportation. Previous literature has found that more walkable environments are associated with more physical activity and less obesity [28, 40]. In addition, the number of fast food restaurants in the U.S has dramatically increased during these study periods; in 2015, there were an estimated 160,000 establishments in the U.S. and 50 million customers served each day [41, 42]. Previous studies have found that greater availability of healthy foods is associated with better diet quality [43] and that the presence of fast food establishments may be associated with more obesity [44, 45]. The association between the physical environment and chronic disease is complex since it involves the interplay of the physical environment and individual behaviors.

The increase in diabetes over the study periods is likely associated with the concomitant observed increase in obesity, the most important risk factor for type 2 diabetes [46]. Previous work suggests that body mass index was the greatest contributor to the change in the prevalence of diabetes between 1976 and 2010 after adjustment for age and race/ethnicity [47]. In addition, the increase in diabetes could be due to the aging U.S. population because diabetes is more prevalent in older adults; the percentage of adults age 36–49 years increased over time in the current study [48]. Furthermore, mortality rates among persons with diabetes have declined, contributing likely to an increase in prevalence [49]. Finally, the U.S. Hispanic population has increased six-fold since 1970 and this race/ethnic group has one of the highest prevalences of diabetes [48, 50]. However, in our study, we found a temporal association for diabetes even after adjusting for age, race, and BMI which suggests that there are other contributing factors. Nevertheless, early-onset diabetes in young adults may result in more cardiovascular complications in older adulthood and the increase, regardless of contributing factors, is important to recognize [51].

A strength of this study was the nationally representative sample of U.S. adults; thus, results can be generalized to the U.S., non-institutionalized population. The consistency of methods in the NHANES allowed for examining data from several decades ago. This study utilized a variety of objective measures from the examination and, therefore, did not rely on self-report for most major outcomes. One exception is CVD which was based solely on self-report. Therefore, it is possible that changes in screening or treatment over time may have affected the reported prevalence of CVD. A limitation of this study is that reliable blood pressure measures and self-report of cholesterol medications were not available in NHANES I and II, therefore, we could only make comparisons to NHANES III. We used one blood pressure cut-point to assess changes in hypertension which allowed us to compare shifts in the distribution of blood pressure measures. Although the cut-points to define hypertension have changed over time for both the general population and for those with specific conditions (e.g., diabetes) we do not expect these changes would impact our conclusions. In addition, blood pressure measures were only collected on one day which could have inflated the prevalence of hypertension. Since the purpose of the study was to compare hypertension over time, we do not expect this limitation to affect our temporal conclusions. Type 1 and type 2 diabetes could not be distinguished in NHANES I or II and diabetes type is difficult to distinguish in NHANES III and the continuous NHANES; thus, as is done in many NHANES studies and reports, the definition of diabetes included both type 1 and type 2. Changes in physical activity, which could influence the health outcomes, could not be assessed since measures were too different across surveys to include as a covariate in this study. Only total cholesterol was available across all study periods, thus, assessment of high-density lipoprotein or low-density lipoprotein was not possible. Analyses could not be stratified by smaller age groups due to the sample size and low prevalence of many health conditions in this relatively young sample population.

Despite increased knowledge about the prevention of chronic disease, the health of younger adults in the U.S. has shown minimal improvements over the past several decades. More adults age 20–49 years are obese and have diabetes and, although the likelihood of uncontrolled hypertension and uncontrolled high cholesterol have generally decreased, more are using hypertensive and cholesterol medications to control these morbidities. These poorer health outcomes can lead to decreased quality of life and economic detriment. At the population level, results from this study should alert physicians, other healthcare workers, and economists about the future implications that the health of younger adults will have on healthcare utilization, the workforce, and economic costs.

## Supporting Information

S1 Table Definitions of health outcomes and the years reported, NHANES 1971–2012. (DOCX)

S2 Table Odds ratio (95% confidence interval) of health outcomes associated with study period among adults age 20–49 years, 1971–2012. (DOCX)
