# Supplementary material for: Cardiovascular Risk Factors of Adults Age 20–49 Years in the United States, 1971–2012: A Series of Cross-Sectional Studies
Source: PLoS One. 2016 Aug 23;11(8):e0161770. doi: 10.1371/journal.pone.0161770 (PMC4995093; doi:10.1371/journal.pone.0161770)
Supplement: S1 Table — (DOCX) [file pone.0161770.s001.docx]

S1 Table. Definitions of health outcomes and the years reported, NHANES 1971-2012

|  | **Years Reported** | **Measure** |
| --- | --- | --- |
| Obesity | 1971-2012 | BMI ≥30.0 kg/m^2^ |
| Overweight | 1971-2012 | BMI 25.0-29.9 kg/m^2^ |
| Ever hypertension | 1988-2012 | BP ≥140/90mmHg or self-report of hypertensive medications |
| Uncontrolled hypertension | 1988-2012 | BP ≥140/90 mmHg |
| Ever high cholesterol | 1988-2012 | Total cholesterol ≥200 mg/dL or self-report of cholesterol medications |
| Uncontrolled high cholesterol | 1988-2012 | Total cholesterol ≥200 mg/dL |
| Diabetes (self-report) | 1971-2012 | Self-report of a physician diagnosis |
| Diabetes (self-report or FPG≥126 mg/dL) | 1976-2012 | Self-report of a physician diagnosis or FPG ≥126 mg/dL |
| Chronic kidney disease | 1988-2012 | eGFR <60 mL/min per 1.73 m^2^ |
| Cardiovascular disease | 1971-2012 | Self-report of heart attack or heart failure |

BMI, body mass index

BP, blood pressure

FPG, fasting plasma glucose

eGRF, estimated glomerular filtration rate; Chronic Kidney Disease Epidemiology Collaboration equation
