# Supplementary material for: Cardiovascular Risk Factors of Adults Age 20–49 Years in the United States, 1971–2012: A Series of Cross-Sectional Studies
Source: PLoS One. 2016 Aug 23;11(8):e0161770. doi: 10.1371/journal.pone.0161770 (PMC4995093; doi:10.1371/journal.pone.0161770)
Supplement: S2 Table — (DOCX) [file pone.0161770.s002.docx]

S2 Table. Odds ratio (95% confidence interval) of health outcomes associated with study period among adults age 20-49 years, 1971-2012

|  | Obesity^*^ | Overweight^†^ | Ever Hypertension^‡^ | Uncontrolled Hypertension^§^ | Ever High Cholesterol^\|\|^ | Uncontrolled High Cholesterol^¶^ | Diabetes (self-report) | Diabetes (self-report or FPG≥126 mg/dL) | CKD** | CVD†† |
| --- | --- | --- | --- | --- | --- | --- | --- | --- | --- | --- |
| NHANES I (1971-1975) | 1.00 | 1.00 |  |  |  |  | 1.00 |  |  | 1.00 |
| NHANES II (1976-1980) | 1.03  (0.89, 1.19) | 0.93  (0.83, 1.04) |  |  |  |  | **1.78**  **(1.22, 2.62)** | 1.00 |  | 0.61  (0.37, 1.00) |
| NHANES III  (1988-1994) | **1.88**  **(1.62, 2.19)** | **1.16**  **(1.04, 1.30)** | 1.00 | 1.00 | 1.00 | 1.00 | **2.13**  **(1.41, 3.22)** | **2.16**  **(1.19, 3.91)** | 1.00 | 1.03  (0.66, 1.60) |
| NHANES 1999-2004 | **3.53**  **(3.09, 4.03)** | **1.61**  **(1.44, 1.81)** | **1.29**  **(1.10, 1.52)** | 1.10  (0.91, 1.32) | 1.05  (0.94, 1.17) | 1.01  (0.91, 1.13) | **2.94**  **(2.04, 4.24)** | **3.42**  **(1.91, 6.14)** | 1.16  (0.55, 2.42) | 0.97  (0.67, 1.43) |
| NHANES 2005-2008 | **4.19**  **(3.54, 4.97)** | **1.75**  **(1.56, 1.96)** | **1.32**  **(1.10, 1.58)** | 0.94  (0.80, 1.12) | 1.02  (0.90, 1.16) | 0.94  (0.83, 1.06) | **3.54**  **(2.43, 5.17)** | **3.11**  **(1.75, 5.52)** | 1.20  (0.53, 2.68) | 1.15  (0.80, 1.66) |
| NHANES 2009-2012 | **4.57**  **(3.86, 5.40)** | **1.81**  **(1.57, 2.09)** | **1.24**  **(1.06, 1.45)** | **0.83**  **(0.70, 0.99)** | 0.95  (0.85, 1.05) | **0.87**  **(0.77, 0.98)** | **3.34**  **(2.26, 4.94)** | **3.50**  **(1.95, 6.29)** | 1.37  (0.65, 2.89) | 1.16  (0.75, 1.79) |

**Bold** text indicates significant estimate

Adjusted for age, sex, and race

^*^ BMI≥30.0kg/m^2^ vs. BMI 18.5-<25.0kg/m^2^

^†^ BMI 25.0-29.9kg/m^2^ vs. BMI 18.5-<25.0kg/m^2^

^‡^ Ever hypertension defined as self-reported hypertensive medication or BP≥140/90 mmHg

^§^ Uncontrolled hypertension defined as BP≥140/90 mmHg

^||^ Ever high cholesterol defined as self-reported cholesterol medication or ≥200mg/dL

^¶^ Uncontrolled high cholesterol defined as ≥200mg/dL

^**^ Chronic Kidney Disease determined by eGRF<60 mL/min per 1.73m^2^ (based on the Chronic Kidney Disease Epidemiology Collaboration Equation

^††^ Cardiovascular disease is self-reported
